# Pernicious anemia in case of chronic viral hepatitis infection: Pernicious anemia and chronic viral hepatitis

**Published:** 2011-03-01

**Authors:** Emmanuel Andres, Mustapha Mecili, Ecaterina Ciobanu

**Affiliations:** 1 Department of Internal Medicine, Diabetes and Metabolic Disorders, University Hospital of Strasbourg, Strasbourg, France

**Keywords:** Viral hepatitis, Hepatitis C, Hepatitis B, Interferon-alpha, Pernicious anemia, Cobalamin deficiency

Dear Editor,

Leonardi and Rosa's (2010) research on the link between celiac disease and viral hepatitis [1] is well designed and focuses on a very interesting topic. Several de novo or latent autoimmune disorders or diseases have been reported in cases of chronic viral hepatitis infections, especially Hepatitis C [2]. In this paper, we report 5 cases of established pernicious anemia during chronic viral hepatitis infection. These cases were extracted from a retrospective (1996 to 2010) study of 82 cases of pernicious anemia in the University Hospital of Strasbourg, France, a tertiary reference center (personal communication in the French Congress of Hematology, Paris, December, 2010). Clinical characteristics of the 5 patients are described in table 1, particularly details on hepatitis infection, modalities of the interferon-alpha treatment, and data on the cobalamin deficiency related to the pernicious anemia. Data of 3 patients were previously published in the literature [3]. It is important to note that, in one case, an association was found between pernicious anemia, true celiac disease, and chronic actve Hepatitis C infection. All the patients were successfully treated with regular intramuscular cyanocobalamin therapy (despite continuous administration of interferon-alpha in 2 cases). To the best of our knowledge, there are only a relatively small amount of cases (< 10 in our own data) documenting a relationship between symptomatic pernicious anemia and viral hepatitis infections. Our patients had established pernicious anemia with documented cobalamin deficiency (in accordance with recent criteria; [4]). They alsfo have a clear chronic active viral hepatitis infection on liver biopsy (1 case of Hepatitis B and 4 cases of Hepatitis C). Moreover, one of our patients had a well-documented case of celiac disease with malabsorption and antitransglutamise antibodies, partially cured with nutritional restrictions (no gluten intake). The pathogenis (the link) of this association is not well knownf, but the role of the virus or of the interferon-alpha treatment as an immuno-modulatory agent is possible [5]. However in our opinion, the fact that this association is exceptional led to the suspicion of a fortuitous association. Thus before any conclusions can be made, future studies similar to Leaonardi and Rosa (2010) are required. Nevertheless, it is important to be aware of the possibility of the association between pernicious anemia and chronic viral hepatitis infections because recognition of this association can help prevent severe cobalamin deficiency.

**Table 1:** Characteristics of the patients

**Age **(years)**, sex**	**Data on viral hepatitis**	**Data on cobalamin deficiency related to pernicious anemia**	**Other autoimmune disorders**
**52, F**	Chronic active hepatitis B followed since at least 5 years of age with no treatment	Cobalamin deficiency:	Hashimoto’s thyroïd with anti-TPO antibodies a
		• Loss of reflexes	
		• Megaloblastic anemia	
		• Serum vitamin B12 level <130 pg/mL a	
		Pernicious anemia:	
		• Anti-intrinsic factor antibodies a	
		• Autoimmune atrophic gastritis	
**45, M**	Chronic active hepatitis C followed since 5 years of age and treated with interféron-α: 9 MU per week (subcutaneously)	Cobalamin deficiency:	-
		• Medullar combined sclerosis	
		• No hematological abnormality	
		• Serum vitamin B12 level <22 pg/mL a	
		Pernicious anemia:	
		• Anti-intrinsic factor antibodies a	
		• Malabsorption on the de Schilling’s test reversed with intrinsic factor b	
		• Autoimmune atrophic gastritis	
**58, F**	Chronic active hepatitis C treated since 7 months of age with interféron-α: 9 MU per week (subcutaneously)	Cobalamin deficiency:	Asymptomatic cryoglobulinemia
		• Loss of reflexes	
		• Megaloblastic anemia and thrombocytopenia	
		• Serum vitamin B12 level <150 pg/mL a	
		Pernicious anemia:	
		• Anti-intrinsic factor antibodies a	
**82, M**	Mild chronic hepatitis C with no interferon-alpha treatment	Cobalamin deficiency:	Antinuclear antibodies a
		• Sensitive peripheral neuropathy	
		• Megaloblastic anemia	
		• Serum vitamin B12 level <20 pg/mL a	
		Pernicious anemia:	
		• Anti-intrinsic factor antibodies a	
		• Autoimmune atrophic gastritis	
**38, M**	Chronic hepatitis C with no interferon-alpha treatment	Cobalamin deficiency:	Celiac disease with malabsorption (iron, folate and calcium), duodal villous atrophy and antitransglutaminase antibodies a
		• Sensitive peripheral neuropathy	
		• Macrocytosis (MCV = 105 fL)	
		• Serum vitamin B12 level <160 pg/mL a	
		Pernicious anemia:	
		• Anti-intrinsic factor antibodies a	

^a^ Serum vitamin B12 levels were determined by radioimmunoassay (Bayer, New York, NY, USA).

^b^ Schilling’s test was realized with a Dicopac test (Amersham Healthcare, Birmingham UK).

## References

[1] Leonardi S, La Rosa M (2010). Are Hepatitis B Virus and Celiac Disease Linked?. Hepat Mon.

[2] Cacoub P, Renou C, Rosenthal E, Cohen P, Loury I, Loustaud-Ratti V, Yamamoto AM, Camproux AC, Hausfater P, Musset L, Veyssier P, Raguin G, Piette JC (2000). Extrahepatic manifestations associated with hepatitis C virus infection: a prospective multicenter study of 321 patients. Medicine.

[3] Andres E, Loukili NH, Ben Abdelghani M, Noel E (2004). Pernicious anemia associated with interferon-alpha therapy and chronic hepatitis C infection. J Clin Gastroenterol.

[4] Andres E, Vidal-Alaball J, Federici L, Loukili NH, Zimmer J, Kaltenbach G (2007). Clinical aspects of cobalamin deficiency in elderly patients. Epidemiology, causes, clinical manifestations, and treatment with special focus on oral cobalamin therapy. Eur J Intern Med.

[5] Andrès E, Maloisel F, Grunenberger F, Noel E, Kurtz J, Lioure B (2002). Interféron [alpha] et auto-immunité: Résultats préliminaires d'une étude prospective de 60 patients. La Revue de Médecine Interne.

